# A study of the professional development needs of Shiraz high schools’ principals in the area of educational leadership

**Published:** 2015-07

**Authors:** ALIASGHAR HAYAT, BIJAN ABDOLLAHI, HASAN REZA ZAINABADI, HAMID REZA ARASTEH

**Affiliations:** Department of Educational Management, Faculty of Management and Accounting, Kharazmi University, Tehran, Iran

**Keywords:** Professional, Development, Need assessment, Educational leadership

## Abstract

**Introduction:**

The increased emphasis on standards-based school accountability since the passage of the no child left behind act of 2001 is focusing critical attention on the professional development of school principals and their ability to meet the challenges of improving the student outcomes. Due to this subject, the current study examined professional development needs of Shiraz high schools principals.

**Methods:**

The statistical population consisted of 343 principals of Shiraz high schools, of whom 250 subjects were selected using Krejcie and Morgan (1978) sample size determination table. To collect the data, a questionnaire developed by Salazar (2007) was administered. This questionnaire was designed for professional development in the leadership skills/competencies and consisted of 25 items in each leadership performance domain using five-point Likert-type scales. The content validity of the questionnaire was confirmed and the Cronbach’s Alpha coefficient was (a=0.78). To analyze the data, descriptive statistics and Paired-Samples t-test were used. Also, the data was analyzed through SPSS14 software.

**Results:**

The findings showed that principals’ “Importance” ratings were always higher than their “Actual proficiency” ratings. The mean score of the difference between “Importance” and “Actual proficiency” pair on “Organizing resources” was 2.11, making it the highest “need” area. The lowest need area was “Managing the organization and operational procedures” at 0.81. Also, the results showed that there was a statistically significant difference between the means of the “Importance” and the corresponding means on the “Actual proficiency” (Difference of means=1.48, t=49.38, p<0.001).

**Conclusion:**

Based on the obtained results, the most important professional development needs of the principals included organizing resources, resolving complex problems, understanding student development and learning, developing the vision and the mission, building team commitment, understanding measurements, evaluation and assessment strategies, facilitating the change process, solving problems and making decisions. In other words, the principals had statistically significant professional development needs in all areas of the educational leadership. Also, the results suggested that today’s school principals need to grow and learn throughout their careers by ongoing professional development.

## Introduction

As we move into the new millennium, education and schools are facing many challenges (1). In this new school environment, there is increasing recognition of the importance of school leadership in supporting change and providing for educational quality. In fact, school leadership has been identified by a number of researchers as a key element in the effectiveness of school organization (2). Educational leadership improvement is the most viable option to making a tremendous difference in education; otherwise, any attempt to make educational reform will fail. In this respect, Tirozzi (2000) believes that enlightened leadership is required to reform educational performance as well as reach student achievement (1). Given the elaborate functions of schools in the 21th century, principals play a crucial role in building efficient and improved schools (3). With regard to continued emphasis of states on school reform and accountability, numerous researchers link schools improvement to the leadership abilities of principals (4-6). Also, some researchers reported that school leadership significantly contributes to improved teaching and learning (5, 7). It should also be stated that effectively positive leader is a vital factor in any successful organizational institution (8).

Given the important and determining role of today’s school leaders, many have taken it for granted that the job of school principal has become more challenging and important than ever (9, 10). It is a job that demands acquisition of qualities, competencies, skills, and generally speaking, professional standards to get the students learning improved (11). In this regard, however, previous studies indicate that principals don’t have sufficient knowledge and skills to take on leadership (6, 9, 12-15). Many novice principals, in fact, feel they are unable, unprepared, alone, isolated, and don’t receive enough support to effectively lead the school (16). On the other hand, school leaders have often reported that they don’t receive enough training to assume leadership position (17) and are ill-prepared for the challenges (5).

Today’s school principals need to grow and learn throughout their careers to adapt to the changing needs of students and schools. The technical, conceptual and public skills demanded of educational leaders have increased dramatically over the last decade. With the widespread acceptance of the need for schools to improve, it is impossible to ignore the critical needs of school leaders to be more effective at their work. They must receive professional development aiming at helping them to be more effective, knowledgeable and qualified to facilitate continuous improvement (1).

Though leadership development is a recent phenomenon (9), studies indicate that development of school principals can lead to school effectiveness and improvement (6, 7, 9, 12-15,18). For this reason, many countries especially in the developed world have come up with institutions and programs for development of school principals (2).

In these studies, development of those principals is recommended as one of the ways of solving those problems. This will enable them to get the skills, knowledge and attributes to run schools in a professional and effective manner to ensure good teaching and learning practices (7, 9, 12-15). Subsequently, it can lead to attainment of the goals and objectives of education which includes improved learning outcomes (1, 3, 17, 19-21).

Allen (2003) in his study discovered that all principals, regardless of their length of service (novice/experienced) or school context (urban/non-urban), had significant professional development needs related to educational technology in all areas (5).

Salazar (2007) in a study entitled “the professional development needs of rural high school principals” reported that most important professional development needs of principals were promoting team commitment, creating a learning organization, sustaining and motivating for continuous improvement, setting instructional direction and communicating effectively (1).

Bichsel (2008) in his study reported that the top five professional development needs of secondary principals in the southwestern Pennsylvania region include analyzing data, communicating effectively, using research and “best practices”, building team commitment, developing information and data collection strategies (22).

As a result, it is suggested that the professional development needs of principals should be enquired continuously in order to determine the form and content of a curriculum for leadership development. Thus, the aim of this study was to determine the professional development needs of Shiraz high schools principals. 

## Methods

The survey method was applied. The statistical population consisted of 343 principals of Shiraz high schools, of whom 250 persons were selected using Krejcie and Morgan (1978) sample size determination table. Also, the participants were selected by simple random sampling from different high schools of Shiraz.

In order to determine what professional development learning opportunities were needed, a needs assessment was conducted using a questionnaire. The Principal Professional Development Needs Assessment questionnaire was used to obtain information concerning a principal’s self-perception of his/her need (or lack of) for professional development in the leadership skills/competencies to facilitate a comprehensive school improvement. This questionnaire consists of 25 items used to identify the leadership domains which are viewed as critical for success in the principal ship. So in this research we used the Salazar questionnaire (1). Salazar (2007) reported that the content validity of the questionnaire was confirmed and the Cronbach’s Alpha coefficient was 0.84. In addition, in our research a pilot study was conducted to measure the internal consistency; the Cronbach’s Alpha was 0.78. Also, the content validity of the questionnaire was confirmed by experts’ opinions in department of education in Kharazmi University. The questionnaire was divided into two sections: demographic professional profile and leadership performance domains. In the first section, information about the independent variables pertaining to the participants’ demographic characteristics was elicited. The second section consisted of 25 items which asked the participants to rate their perceived level of professional development need in each leadership performance domain using Five-point Likert-type scales. In other words, the survey was designed so that the principals valued 25 items related to leadership performance domains. This is labeled as "Importance" on the survey. The survey was also designed to measure principals’ "Actual Proficiently" on those similar 25 items related to leadership performance domains. The difference (or gaps) between "Importance" and their "Actual Proficiently" was indicative of the principals’ professional development needs.

All descriptive statistics and inferential statistics were performed using SPSS 14. To analyze the data, we measured the principals’ "Actual Proficiently and importance on those similar 25 items related to leadership performance domains, using Mean and SD. Deviation. Also, Paired-Samples t-test was performed to investigate the difference (or gaps) between "Importance" and "Actual Proficiently” of the principals in leadership performance domains. 

## Results

According to descriptive indicators, 44% and 56% were female and male, respectively. Also 0.4% of the sample had a diploma degree, 21.6% associate degree, 56.8% bachelor degree and 21.2% master degree.

**Table1:** Mean and standard deviation for importance and actual proficiency surveys items

**Survey Item**	Item	Mean±SD	Difference of means ("Importance"- "Actual proficiency")
**Building team commitment **	Importance	4.51±0.51	1.69
	Actual proficiency	2.82±0.38	
**Creating a learning organization **	Importance	4.07±0.85	1.20
	Actual proficiency	2.87±0.60	
**Developing and implementing strategic action plans **	Importance	3.95±0.26	1.33
	Actual proficiency	2.62±0.57	
**Understanding measurements, evaluation and assessment strategies **	Importance	4.73±0.23	1.83
	Actual proficiency	2.90±0.47	
**Communicating effectively **	Importance	4.50±0.52	1.31
	Actual proficiency	3.19±0.85	
**Facilitating the change process **	Importance	4.68±0.46	0.70
	Actual proficiency	2.98±0.55	
**Building shared decision making, collegiality and peer support **	Importance	4.69±0.23	1.47
	Actual proficiency	3.22±0.71	
**Using research and "best practice" **	Importance	4.51±0.51	1.69
	Actual proficiency	2.82±0.95	
**Understanding student development and learning **	Importance	4.49±0.50	1.94
	Actual proficiency	2.55±0.66	
**Resolving complex problems **	Importance	4.52±0.50	1.91
	Actual proficiency	2.43±0.64	
**Solving problems and making decisions **	Importance	4.69±0.47	1.78
	Actual proficiency	2.91±0.77	
**Building community and involvement **	Importance	4.01±0.80	1.18
	Actual proficiency	2.83±0.71	
**Building consensus and negotiating effectively **	Importance	3.98±0.79	1.10
	Actual proficiency	2.88±0.78	
**Facilitating professional development/development of others **	Importance	3.99±0.81	0.85
	Actual proficiency	3.14±0.56	
**Setting instructional direction - results orientation **	Importance	4.49±0.52	1.59
	Actual proficiency	2.90±0.43	
**Setting goals and determining outcomes **	Importance	4.18±0.76	1.44
	Actual proficiency	2.74±0.64	
**Developing the vision and the mission **	Importance	4.50±0.51	1.96
	Actual proficiency	2.54±0.62	
**Designing, implementing, and evaluating curriculum **	Importance	4.51±0.50	1.75
	Actual proficiency	2.76±0.67	
**Defining the core values and beliefs of education **	Importance	4.28±0.71	1.70
	Actual proficiency	2.58±0.74	
**Analyzing data **	Importance	3.68±0.98	0.80
	Actual proficiency	2.88±0.91	
**Developing information and data collection strategies **	Importance	4.35±0.65	1.39
	Actual proficiency	2.96±0.74	
**Sustaining and motivating for continuous improvement **	Importance	4.76±0.42	1.33
	Actual proficiency	3.43±0.88	
**Developing the school organization using systems thinking **	Importance	4.00±0.81	0.94
	Actual proficiency	3.06±0.89	
**Managing the organization and operational procedures **	Importance	3.99±0.82	0.71
	Actual proficiency	3.18±0.84	
**Organizing resources**	Importance	4.74±0.45	2.11
	Actual proficiency	2.63±0.58	
**Leadership performance (Total )**	Importance	4.35±0.42	1.48
	Actual proficiency	2.87±0.20	

Table 1 presents the descriptive statistics for all of the items on the survey. This table is particularly important not only because it displays the mean scores for all of the items on the survey, but also because it shows that in every pair of questions “Importance” is always rated higher than “Actual proficiency.” SPSS was used to calculate the means and standard deviations for individual survey items on each of the “Importance” and “Actual proficiency” domains of the survey instrument. In this instance, it was important to analyze which individual items appeared to present the greatest “need” for professional development. Means of the responses to the items on the “Importance” domains ranged from a maximum of 4.76 on Question 22 which measured “Sustaining and motivating for continuous improvement”, to a minimum of 3.68 on Question 20 which measured “Analyzing data”. Means of the responses to items on the “Actual proficiency” domains ranged from a maximum of 3.43 on Question 22 which measured “Sustaining and motivating for continuous improvement” to a minimum of 2.43 on Question 10 which measured “Resolving complex problems”.

The difference between “Importance” and “Actual proficiency” on the instrument represented the professional development needs of principals. As noted in the previous section, in every case, principals’ “Importance” ratings were always higher than their “Actual proficiency” ratings. This difference, for the purposes of the study, constituted their professional development needs in the area of leadership performance domains. To test the significance of these differences, a paired sample t-test was used. In this instance, the t-test was used to determine if there were significant differences among the means of the “Importance” and “Actual proficiency” of the instrument. The results showed that there was a statistically significant difference between the means of the “Importance” and the corresponding means on the “Actual proficiency” (t= 49.38, p<0.001) (Table 2).

**Table 2:** T-test results for the difference between “Importance” and “Actual Proficiency

	Difference of Means ("Importance"- "Actual proficiency")	SD Deviation	SD Error Mean	t	df	p
**Leadership performance domains**	1.48	0.475	0.030	49.38	249	<0.001

## Discussion

1. Principals highly value all of the expected areas of performance as indicated by their ratings on the “Importance” domains of the Educational leadership for Principals Survey. It was concluded that principals highly value each of the standards.

2. Principals’ responses to “Actual proficiency “on all of the survey items were always rated lower than their responses to the corresponding “Importance” on the items. Also the results showed that there was a statistically significant difference between the means of the “Importance” and the corresponding means on the “Actual Proficiency”. This difference suggests that principals have a need for professional development on each standard. 

These structures highlighted the fact that professional development activities should be unique to the individual principal. Depending on whether a principal is novice or experienced, elementary or secondary, serving in a suburb or in an urban district, it is clear that the focus must be on the unique needs of individual principals. The rationale for conducting a needs assessment was the final section of the background and related literature. Part of the reason for the minimal attention on the topic of professional development for principals has been a lack of understanding of what principals should know and be able to do in this area.

The results of this study revealed that the principals are concerned about the leadership needed for school improvement. They stated they needed more professional development in order to meet the new expectations of their role. A large proportion of the principals perceived that they lacked the skills to build the collaborative learning organization that is so critical to successful school improvement. Clearly, principals must be provided with quality professional development if schools are going to successfully serve every student (1, 2, 7, 8, 16).

Good leadership is not innate (1). The main leadership challenges to which the principals are faced today are organizational. Leaders must be able to establish expectations on the norms of teaching and learning for all members of the learning community while building organizational systems to support them and maintaining a professional climate that encourages practitioners to continue to learn. Leadership today requires the ability to mobilize the constituents to do important but difficult work under conditions of constant change, overload, and fragmentation. This requires ongoing professional development opportunities to help the principals update their leadership knowledge and skills on an ongoing basis (10-13).

## Conclusion

The findings show that the principals are in a poor condition with regard to all key areas. To explore why it is so, we can return to the literature demonstrating that the leaders aren’t appropriately trained to take on the job of school principalship and that they are ill-prepared for the existing challenges. Also, leaders have frequently stated that trainings provided by universities don’t get them ready for school leadership. In addition, many scholars are dubious about the appropriateness of conventional programs for getting the principals equipped. Pashiard and Brauckmann argued that principals, in some regions, are assigned based on inappropriate criteria, such as teaching experience rather than leadership skills, experience and irrelevant academic qualifications, and even favoritism. In many cases, teachers are also assigned principals without having adequate training. It can be, therefore, stated these factors altogether have caused principals to feel incapable in their new positions; hence, as mentioned earlier, professional development through needs assessment provides the principals with knowledge, attitude, and adequate competencies so as they will effectively and professionally lead their schools which, subsequently, help to realize education goals and purposes. 
